# Clinicopathological and molecular markers associated with prognosis and treatment effectiveness of endometrial stromal sarcoma: a retrospective study in China

**DOI:** 10.1007/s00404-013-2987-5

**Published:** 2013-08-20

**Authors:** Li He, Jun-Dong Li, Ying Xiong, Xin Huang, Long Huang, Jia-xin Lin, Yun Zhou, Min Zheng

**Affiliations:** Department of Gynecology, State Key Laboratory of Oncology in Southern China, Sun Yat-sen University Cancer Center, 651 Dongfeng Road East, Guangzhou, Guangdong 510060 People’s Republic of China

**Keywords:** Endometrial stromal sarcoma, Pelvic lymphadenectomy, CD10, Prognosis

## Abstract

**Purpose:**

To evaluate the clinicopathological and immunophenotypic characteristics of endometrial stromal sarcoma (ESS) in China.

**Methods and materials:**

Seventy-two consecutive ESS cases treated between 1995 and 2009 were retrospectively reviewed.

**Results:**

Sixty-three patients received surgical treatment. Forty-one patients underwent pelvic lymphadenectomy. In paraffin-embedded specimens, expression of the following molecular markers was detected: CD10 (27/36), vimentin (37/38), HHF35 (3/32), S-100 (0/25), desmin (2/29), CD117 (0/23), CD34 (2/24), alpha-inhibin (0/17), CK (1/34), CD99 (4/9), smooth muscle actin (5/25), EMA (0/7), estrogen receptor (13/16) and progesterone receptor (13/16). CD10 and vimentin were expressed more frequently in these specimens. Tumor classification, CD10 and surgical procedures were significantly associated with disease-free survival (DFS). Surgical procedures were significantly associated with overall survival (OS). Tumor stage (*P* = 0.024) and surgical procedure (*P* = 0.042) were found to be significant independent prognostic factors for DFS. No complete or partial response was observed among patients who received radiotherapy or chemotherapy.

**Conclusions:**

Our results indicate that total hysterectomy with bilateral salpingo-oophorectomy followed by pelvic lymphadenectomy is associated with an improved treatment outcome. CD10-negative expression may contribute to the malignant characteristics and recurrence associated with ESS.

## Introduction

Endometrial stromal sarcoma (ESS) is a rare uterine malignancy that accounts for <0.5 % of all malignant uterine tumors and for 7–17 % of uterine sarcomas [1, 2]. Traditionally, ESS is classified into low- and high-grade subtypes based on its mitotic rate. Currently, ESS is divided into low-grade endometrial stromal sarcomas and undifferentiated endometrial sarcoma (UES) according to the 2003 World Health Organization Classification of Tumors [3]. Its rarity and histological differences underlie the difficulty in diagnosing ESS and the choice of subsequent treatment strategies. Factors such as mitotic index, size, clinical stage, histological grade, positive surgical margins, menopause and age have been reported as potential prognostic parameters, but their use in ESS remains controversial [4, 5].

CD10, or commonly known as acute lymphoblastic leukemia antigen, is a cell-surface neutral endopeptidase that deactivates bioactive peptides [6, 7]. Recently, CD10 has been demonstrated to be present within a variety of non-hematopoietic neoplasms, including the normal endometrial stroma and ESS [8–10]. To identify possible molecular markers that correlate with prognosis of ESS patients, we analyzed retrospectively the immunohistochemical results of ESS patients.

This retrospective study aimed to evaluate the clinical characteristics, immunophenotype, diagnosis, management, and prognosis of ESS patients who were treated at the Cancer Center, Sun Yat-sen University (Guangdong, China) over a 15-year period.

## Materials and methods

The Review Board of our Cancer Center of the Sun Yat-sen University approved this study. All consecutive cases of ESS diagnosed from 1995 to 2009 at the Cancer Center, Sun Yat-sen University were reviewed. Data were retrieved from medical records maintained in institutional databases. The study only included patients diagnosed with ESS who had been histologically confirmed based on WHO classification, and staged according to the International Federation of Gynecology and Obstetricsthe (FIGO) classification (cases confirmed in foreign hospitals were all taken for consultation with gynecological pathologists from Sun Yat-sen University). Pathological diagnosis was conducted by experienced pathologists of Sun Yat-sen University.

For patients who underwent complete resection, the surgical procedure generally comprised total abdominal hysterectomy with bilateral salpingo-oophorectomy and pelvic lymphadenectomy. For incomplete resection patients (partial or complete hysterectomy), bilateral salpingo-oophorectomy and pelvic lymphadenectomy were carried out. For patients suspected of common iliac or para-aortic lymph node involvement, para-aortic lymph node dissection was performed. Omentectomy was administered for stage III ESS patients.

For patients with one of the following pathological risk factors: incomplete resection for late-staged cases, positive pelvic lymph nodes, vascular and lymphatic permeation and UES, adjuvant radiotherapy and/or adjuvant chemotherapy was administered. The radiotherapy treatment comprised external pelvic irradiation (18 MV X-rays) using the multiportal technique; in one fraction of 1.8–2.0 Gy daily, for a total dose of 50 Gy over 5–6 weeks.

Chemotherapy comprised one of the following combinations as the primary drug for treatment: DDP (cisplatin) (60–75 mg/m^2^, d1) + cyclophosphamide (600–800 mg/m^2^, d1-3) + adriamycin (40–50 mg/m^2^, d1), DDP (60–75 mg/m^2^, d1) + adriamycin (40–50 mg/m^2^, d1) + DTIC (dacarbazine) (200 mg/m^2^, d1-5), or ifosfamide (IFO) (1.5–2 g/m^2^, d1-4) + adriamycin (40–50 mg/m^2^, d1) + Dacarbazine (DTIC) (200 mg/m^2^, d1-5). Dosage was decreased when IV degree bone marrow suppression occurred. In general, chemotherapy was performed in 3–5 courses over a 3-week period.

For each patient included in this study, the following demographic and clinical data (last follow-up performed in February, 2012) were obtained and listed: symptoms, age, menopausal status, parity, family history of cancer, diagnosis procedure, tumor stage, surgical procedure, pathological diagnosis, tumor size, lymphatic vascular space invasion, depth of invasion, recurrent/metastatic sites, chemotherapy history, radiotherapy history and hormonal therapy history. Individual diagnosis for each case was based on postoperative pathological results. Where recurrence was suspected, the corresponding patient’s diagnosis was subject to identification by chest X-rays, a CT scan of the chest and/or brain, and a PET-CT on the basis of a physical examination. The time of diagnosis was defined as the date of primary surgical procedure. The median duration of follow-up was 61.6 months (range, 15–185.6 months). OS was defined as the interval from surgery to death from any cause. Status of patients was confirmed at the date of last contact. DFS was defined as the time interval from surgical resection to the first evidence of recurrence or death from any cause, whichever occurred first. Patients were examined at 2-month intervals for the first 2 years, at 6-month intervals for the next 3 years and, thereafter, once a year. The primary end point was any cancer-related death.

For immunostaining, 2 μm sections of tissue were cut and mounted on glass slides. The sections were heated at 60 °C for 2 h and deparaffinized in xylene and ethanol. Antigen retrieval was performed using EDTA buffer (1 mmol/L, pH 8.0) and boiled for 20 min in a microwave oven, followed by treatment with 3 % H_2_O_2_ to block endogenous peroxidase for 15 min at room temperature. The slides were incubated at 4 °C overnight antibody. Antibody staining was done using ChemMateTM EnVisionTM Detection Kit, Peroxidase/DAB, Rabbit/Mouse (GeneTech, Shanghai, China). The sections were incubated with horseradish peroxidase-conjugated secondary antibody (Zhong-shan Golden Bridge Biotech, Beijing, China) for 30 min at room temperature. Hematoxylin was used for counterstaining. Technical personnel of the pathology department in our center performed all the immunohistochemistry experiments. Two pathologists confirmed all the results in a double-blind analysis. The pathological results from the medical record library were further analyzed.

DFS and OS were estimated with the Kaplan–Meier method and were compared by a log-rank test using GraphPad Prism software (version 5, GraphPad Software). Cox regression analysis of factors, including age, tumor classification, tumor stage, lymphovascular space invasion, depth of invasion, adjuvant therapy, surgical procedure and CD10 expression, which were potentially related to survival, was used to identify independent factors that might jointly have a significant effect on survival. All statistical analyses were performed with SPSS 16.0 software. A *P* value <0.05 was considered significant.

## Results

### Patient characteristics

Seventy-nine consecutive ESS patients were treated at Sun Yat-sen University, among which nine patients were excluded because of insufficient follow-up data (for details, see Tables 1, 2), and seven were excluded because they were not diagnosed with ESS at the time immediately post-transfer to Sun Yat-sen University following a non-standard primary operation conducted at a foreign hospital. Among the 63 cases taken for study, 50 (79.4 %) were treated primarily at Sun Yat-sen University (including 29 patients with complementary operation), and 13 (20.6 %) were either referred to or were undergoing adjuvant therapy at Sun Yat-sen University after the primary operation (with a total hysterectomy baseline) and other adjuvant therapy. Patients’ clinical and histopathological characteristics are summarized in Table 3. The median age at diagnosis was 41.1 years (range: 19–61 years). Abnormal bleeding (55.6 %) and pain (17.5 %) were the most frequent symptoms. Fifteen (23.8 %) patients were nulliparous and nine (14.3 %) were postmenopausal at the time of diagnosis. Thirteen (20.6 %) patients were preoperatively diagnosed with ESS by dilation and curettage or other methods; the remaining patients were diagnosed in or after surgery. The median tumor size was 5.3 cm (range 2–11 cm) with relevant data missing in 22 (34.9 %) patients. 51 (80.9 %) patients were diagnosed as having low-grade ESS and 6 (14.6 %) as having UES. Within these 63 cases, the majority (63.5 %) of patients’ disease stage was in stage I, 6.3 % stage II, 25.4 % stage III, and 4.8 % stage IV.

**Table 1:** General characteristics and treatments of 9 excluded ESS patients

Case	Initial treatment (other hospitals)					Replacement therapy (our hospital)		
	Age	Treatment	Pathology	Recurrence time (months)	Recurrence	Treatments	Pathology	Follow-up
1	42	Subtotal hysterectomy	ESS	33	Vagina, lung	Un-completed chemotherapy	No	No
2	51	TAH	ESS	3	Pelvic	Untreated	No	No
3	46	Subtotal hysterectomy	Endometrial adenomatous hyperplasia	15	Pelvic	Untreated	No	No
4	47	Subtotal hysterectomy	Adenomyomatosis	39	Pelvic	Pelvic resection + omentectomy	Omental adenocarcinoma	No
5	35	TAH	Unknown	18	Vagina	Vaginal tumor biopsy	ESS	No
6	45	TAH	Unknown	92	Pelvic	Palliative operation and chemotherapy	ESS	No
7	31	Subtotal hysterectomy	Unknown	41	Pelvic, vagina	Uncompleted chemo-radiotherapy	ESS	No
8	35	Subtotal hysterectomy	ESS	60	Pelvic, lung	Uncompleted chemotherapy	No	No
9	39	Palliative operation	UES	1		Palliative operation	No	No

**Table 2:** Expression of genes in 9 excluded ESS patients

Case	CD10	CK	VIM	SMA	EMA	HHF35	S100	CD117	Desmin	CD34	ER	PR	KI-67
1													
2													
3													
4	0	0	1	0		0	0	1	0	0	1	1	
5			1	0			0			0			
6	0	1	1		1	0	0	1		0			
7													
8	1												
9	1		1		0		0	0	0	0	0	0	10 %

**Table 3:** Patients’ clinical and histopathologic characteristics (*n* = 63)

Characteristics	No. of patients (%)
Tumor’s classification	
Low-grade ESS	51 (80.9)
UES	6 (9.5)
Unknown	6
Menstruation status	
Premenopausal	54 (85.7)
Postmenopausal	9 (14.3)
Parity	
Multiparous	48 (76.2)
Nulliparous	15 (23.8)
Diagnosis procedure	
Diagnostic dilatation and curettage	9 (14.3)
Hysteroscopic biopsy	2 (3.17)
Vaginal/cervical tumor biopsy	2 (3.17)
Intraoperative frozen	12
Tumor stage	
I	40 (63.5)
II	4 (6.3)
III	16 (25.4)
IV	3 (4.8)
Depth of invasion	
Mucous invasion	4 (6.3)
Myometrium	40 (63.5)
<½ myometrium	5
>½ myometrium	14
Myometrium (unknown depth)	21
Serosal invasion	5 (7.9)
Unknown	14
LVSI	
Yes	11 (17.5)
No	27 (42.9)
Unknown	25
Surgical procedure	
TAH ± BSO/OT/PL/PLND	11 (17.5)
TAH + BSO ± /PL/OT	13 (20.6)
TAH + BSO + PL ± OT ± PLND	39 (61.9)
Hysterectomy	
With BSO	52 (82.5)
Without BSO	11 (17.5)
Pelvic lymphadenectomy	
Yes	41 (65.1)
No	22 (34.9)
Postoperative adjuvant therapy	
Chemotherapy	48 (76.2)
Radiotherapy	15 (23.8)
Hormonal therapy	3 (4.7)
Chemotherapy and radiotherapy	11 (17.5)
Chemotherapy and hormonal therapy	3 (4.8)
Radiotherapy and hormonal therapy	1 (1.6)
No adjuvant therapy	11 (17.5)
Distant metastasis and recurrence	13 (20.6)
Pelvic	6
Lung and bone	2
Pelvic and lung	5

*TAH* total abdominal hysterectomy, *BSO* bilateral salpingo-oophorectomy, *PL* pelvic lymphadenectomy, *OT* omentectomy, *PLND* para-aortic lymph node dissection, *LVSI* lymphatic vascular space invasion

## Surgical procedure

Thirty-nine patients (61.9 %) underwent a total hysterectomy with bilateral salpingo-oophorectomy, and pelvic lymphadenectomy ± omentectomy/para-aortic lymph node dissection, 13 patients (20.6 %) had a total hysterectomy with bilateral salpingo-oophorectomy ± /para-aortic lymph node dissection/omentectomy, and 11 patients (17.5 %) had a total hysterectomy ± bilateral salpingo-oophorectomy/pelvic lymphadenectomy/para-aortic lymph node dissection/omentectomy, as part of surgical treatment.

Fifty-two (82.5 %) patients had a bilateral salpingo-oophorectomy and 11 (17.5 %) did not; 41 (65.1 %) patients had a pelvic lymphadenectomy and 22 (34.9 %) did not.

**Fig. 1:**
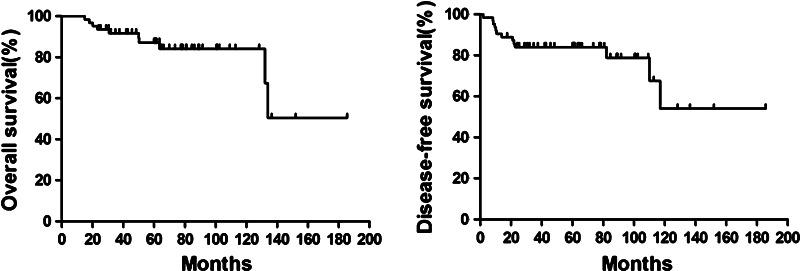
Survival curves for overall survival (OS) and disease-free survival (DFS) associated with 63 patients diagnosed with endometrial stromal sarcoma ESS

After primary surgery, 52 patients (82.5 %) received adjuvant therapy, 15 (23.8 %) were administered pelvic radiation at 50 Gy, 48 (76.2 %) received chemotherapy, 11 (17.5 %) received chemotherapy plus radiotherapy, three (4.8 %) received chemotherapy plus hormonal therapy, and one (1.6 %) received radiotherapy plus hormonal therapy. The 2-year DFS rate was 82.5 %, and the 5-year DFS rate was 53.9 %. The median DFS duration was 53.8 months. The 2-year OS rate was 93.6 %, and the 5-year OS rate was 60.3 %. The DFS and OS curves are shown in Fig. 1.

## Recurrences

Recurrence developed in 13 (20.6 %) of the 63 patients, resulting in 10 (15.7 %) reported deaths at the time of last follow-up, with locations of recurrence as follows: pelvic recurrence (6/13, 46.1 %), extrapelvic recurrence (2/13, 15.4 %) and both pelvic and extrapelvic recurrences (5/13, 38.5 %). Analysis of clinical data revealed that patients diagnosed as having UES exhibited a significantly shorter DFS (16.8 vs. 86.3 %; *P* = 0.0007) and OS (50 vs. 88.2 %; *P* = 0.0234) compared to those having low-grade ESS. The 19 (30.2 %) patients in disease stages III–IV showed a shorter DFS (68.4 vs. 84.1 %; *P* = 0.06) and OS (73.7 vs. 88.6 %; *P* = 0.0744) than the remaining 44 (69.8 %) patients with stages I–II. Among the 63 patients, 41 (65.1 %) had previously undergone pelvic lymphadenectomy, and six (9.5 %) were found to have nodal metastases. Kaplan–Meier survival analyses showed that patients who did not undergo a bilateral salpingo-oophorectomy had a significantly shorter DFS (45.5 vs. 86.5 %; *P* = 0.0169) and OS (54.5 vs. 90.4 %; *P* = 0.0475) than those who received a bilateral salpingo-oophorectomy. Patients who did not undergo a pelvic lymphadenectomy showed a similar association with shorter DFS (54.5 vs. 92.7 %, *P* = 0.001) and OS (63.6 vs. 95.1 %; *P* = 0.0025) than patients who did receive a pelvic lymphadenectomy. Patients who underwent a total hysterectomy with bilateral salpingo-oophorectomy and pelvic lymphadenectomy displayed a significantly longer DFS (92.3 vs. 58.3 %, *P* = 0.0038) and OS (94.9 vs. 66.7 %; *P* = 0.023) than patients undergoing total hysterectomy ± salpingo-oophorectomy/pelvic lymphadenectomy/para-aortic lymph node dissection/omentectomy only. By contrast, patient age, menopausal status, parity, tumor size, lymphatic vascular space invasion, depth of invasion, and adjuvant therapy did not show any significant association with DFS or OS.

## Molecular expression

For a subset of 46 cases, additional immunohistochemical assays had been performed to detect the expression of certain proteins, including CD10 (27/36), vimentin (37/38), HHF35 (3/32), S-100 (0/25), desmin (2/29), CD117 (0/23), CD34 (2/24), alpha-inhibin (0/17), CK (1/34), CD99 (4/9), smooth muscle actin (5/25), EMA (0/7), estrogen receptor (5 for 3+, 4 for 2+, 4 for 1+, 3 for 0), and progesterone receptor (7 for 3+, 1 for 2+, 5 for 1+, 3 for 0). The Ki-67 proliferation index values were also available, and included values of <30 % for 13 cases and ≥30 % for six cases. The DFS values for those who were ki-67 positive (≥30 %) were 50 versus 76.9 % for those who were ki-67 negative (<30 %) (*P* = 0.129) and overall survival was 66.7 vs. 84.6 % (*P* = 0.436), respectively. CD10-negative expression was significantly associated with tumor classification (*P* = 0.001), distant metastasis and recurrence (*P* = 0.003), but was not significantly related to patient age, menopausal status, parity, tumor size, lymphatic vascular space invasion, or depth of invasion (Table 4). Kaplan–Meier survival analyses showed that patients with CD10-negative expression had a shorter DFS (33.3 vs. 88.9 %; *P* = 0.02) and OS (55.6 vs. 88.9 %; *P* = 0.11) than those who were CD10-positive.

**Table 4:** Correlations between CD10 expression and clinicopathological features of ESS patients

Characteristics	Total	CD 10		* *P* value
		Positive (*n* = 27)	Negative (*n* = 9)	
Age (years)				
<50	21	16 (59.3)	5 (55.6)	1.0
≥50	15	11 (40.7)	4 (44.4)	
Menstruation status				
Premenopausal	30	24 (88.9)	6 (66.7)	0.151
Postmenopausal	6	3 (11.1)	3 (33.3)	
Parity				
Multiparous	30	24 (88.9)	6 (66.7)	0.151
Nulliparous	6	3 (11.1)	3 (33.3)	
Tumor size				
≥5 cm	17	14 (77.8)	3 (50)	0.307
<5 cm	7	4 (22.2)	3 (50)	
Tumor’s classification				
Low-grade ESS	28	25 (96.2)	3 (37.5)	0.001
UES	6	1 (3.8)	5 (62.5)	
Tumor stage				
I	22	18 (66.7)	7 (77.8)	0.69
II	3			
III	9	9 (33.3)	2 (22.2)	
IV	2			
Depth of invasion				
Mucous invasion	3	5 (38.5)	3 (50)	1
Myometrium	24			
<½ myometrium	5			
>½ myometrium	8	8 (61.5)	3 (50)	
Serosal invasion	3			
LVSI				
Yes	9	8 (42.1)	1 (25)	1
No	14	11 (57.9)	3 (75)	
Distant metastasis and recurrence				
Yes	9	3 (11.1)	6 (66.7)	0.003
No	27	24 (88.9)	3 (33.3)	

* Two-side Fisher’s exact test

## Univariate and multivariate analysis

Various clinicopathological variables were evaluated to identify potential prognostic factors for survival. In univariate analyses, death from ESS was associated with surgical procedure (hazard ratio, 5.13; 95 % CI, 1.074–14.497; *P* = 0.04). This was also associated with DFS (hazard ratio, 5.48; 95 % CI, 1.504–19.967; *P* = 0.01). Additionally, recurrence from ESS was also associated with tumor’s classification (hazard ratio, 6.047; 95 % CI, 1.925–18.996; *P* = 0.002) and CD10-negative expression (hazard ratio, 4.696; 95 % CI, 1.049–21.015; *P* = 0.043). However, upon multivariate Cox regression analysis (including tumor stage, tumor’s classification and surgical procedure), none of them remained as independent predictors of OS (Table 5). Conversely, tumor stage (*P* = 0.024) and surgical procedure (*P* = 0.042) were found to be significant independent prognostic factors for DFS.

**Table 5:** Cox regression analysis of various factors associated with disease-free survival and overall survival in ESS patients

Disease-free survival			
Variables	HR (95 % CI)	Favorable/unfavorable	*P* value
Univariate analysis			
Age (years)	0.771 (0.237–2.509)	<50/≥50	0.661
Tumor’s classification	6.047 (1.925–18.996)	Low-grade ESS/UES	**0.002**
Tumor stage	2.748 (0.909–8.18)	I–II/III–IV	0.073
LVSI	4.239 (0.707–25.405)	No/yes	0.114
Depth of invasion	2.602 (0.288–23.531)	<½ myometrium/>½ myometrium	0.395
Surgical procedure	5.48 (1.504–19.967)	B/A	**0.01**
Adjuvant therapy	2.349 (0.302–18.282)	No/yes	0.415
CD10	4.696 (1.049–21.015)	Positive/negative	**0.043**
Multivariate analysis			
Tumor’s classification	3.068 (0.881–10.689)	Low-grade ESS/UES	0.078
Tumor stage	3.789 (1.190–12.064)	I–II/III–IV	**0.024**
Surgical procedure	4.489 (1.059–19.022)	B/A	**0.042**

Overall survival			
Variables	HR (95 % CI)	Favorable/unfavorable	*P* value
Univariate analysis			
Age (years)	1.472 (0.408–5.305)	<50/≥50	0.5555
Tumor’s classification	3.139 (0.731–13.48)	Low-grade ESS/UES	**0.124**
Tumor stage	3.091 (0.888–10.759)	I–II/III–IV	0.076
LVSI	4.066 (0.677–24.414)	No/yes	0.141
Depth of invasion	1.963 (0.201–19.175)	<½ myometrium/>½ myometrium	0.562
Surgical procedure	5.13 (1.074–14.497)	B/A	**0.04**
Adjuvant therapy	1.641 (0.204–13.196)	Yes/no	0.642
CD10	3.358 (0.675–16.707)	Positive/negative	0.139
Multivariate analysis			
Tumor’s classification	4.476 (0.962–20.812)	Low-grade ESS/UES	0.056
Tumor stage	2.746 (0.562–13.409)	I–II/III–IV	**0.212**
Surgical procedure	3.136 (0.587–16.748)	B/A	0.181

* Two-side Fisher’s exact test

^A^TAH ± BSO/OT/PL/PLND

^B^TAH + BSO + PL ± OT ± PLND

## Discussion

ESS are rare uterine malignancies that typically affect women between 40 and 50 years of age and manifest clinically through vaginal bleeding, [11] as exemplified in this study, with the average age of the 63 patients being 41.1 years and vaginal bleeding being the most distinctive symptom (55.6 %). Although early symptoms are often observable in patients, many are diagnosed postoperatively because of the decreased sensitivity of endometrial pipelle for detecting ESS (only 13/63 patients were diagnosed with ESS before surgical treatment in our study). Thus, 34/63 patients received a secondary operation following the first (subtotal hysterectomy or total hysterectomy). The surgical modality for ESS remains controversial. Although certain gynecologic oncologists prefer that ESS be staged as endometrial adenocarcinoma [12], some studies have shown that including lymphadenectomy or ovarian preservation does not affect prognosis [13–15]. Conversely, studies have found that recurrence does exist in patients with retained ovaries [16, 17]. This high rate of recurrence following a hysterectomy may in part be caused by estrogen stimulation originating from the retained ovaries. Our data revealed that ovarian removal was effective in delaying recurrences (*P* = 0.0169) and had a significant effect on OS (*P* = 0.0475). ESS continues to be staged according to FIGO guidelines for endometrial cancer; therefore, initial complete staging would necessitate lymph node sampling. A recent American series of 1010 ESS patients showed that adding lymphadenectomy to hysterectomy did not change the OS [18]. However, another recent Canadian series showed a substantial lymph node involvement rate in 33 % of ESS [19]. Cheng [20] found that 22 % of patients who underwent pelvic lymphadenectomy had positive lymph nodes at surgery. In our study, among 63 patients, 41 (65.1 %) previously underwent pelvic lymphadenectomy, and six (9.5 %) were found to have nodal metastases. We found that pelvic lymphadenectomy had a significant advantage both in DFS (*P* = 0.001) and OS (*P* = 0.0025). Patients who underwent total hysterectomy with bilateral salpingo-oophorectomy and pelvic lymphadenectomy ± para-aortic lymph node dissection/omentectomy presented a longer DFS (*P* = 0.0038) and OS (*P* = 0.023), which could additionally act as a significant independent prognostic factor of DFS in ESS (*P* = 0.042). As such, we advocate the integration of pelvic lymphadenectomy and bilateral salpingo-oophorectomy into a standardized operation.

While data support the use of chemotherapy in the treatment of other sarcomas [21], very little data exist to support its use in the treatment of ESS. The European Organisation for Research and Treatment of Cancer Gynaecological Cancer Group Study proposed a trial to evaluate the treatment effect of adjuvant radiotherapy on all uterine sarcoma types. However, this randomized study failed to show any benefit of adjuvant RT in terms of OS and DFS in patients with stage I or II sarcoma [22]. Weitmann [23] previously proposed total hysterectomy and bilateral salpingo-oophorectomy followed by adjuvant radiation therapy as the most effective treatment for patients. Presently, adjuvant hormonal therapy appears to be used predominantly in ESS patients. Patients with primary residual or recurrent ESS were managed with hormonal therapy and showed an 82 % response rate with a median duration of response of 48 months [24, 25]. After comparing the status of patients who received chemotherapy and or radiotherapy or chemotherapy + radiotherapy with those who received no additional treatment in all stages, we determined no association between adjuvant treatment and OS or DFS. Unfortunately, we were unable to analyze cases for patients who accepted hormone therapy after surgery, as outpatient records frequently did not meet the review standards because of the limited number of patients received, and the difficulty of follow-up.

Developments in recent years have shown that immunohistochemistry can be useful, not only in the differential diagnosis, but also in the prognosis of tumors of the female genital tract. Currently, however, no specific immunohistochemical markers exist for the diagnosis of ESS. In our study, the group of ESS cases expressed CD10, vimentin, HHF35, desmin, CD34, CK, CD99, smooth muscle actin, estrogen receptor, and progesterone receptor. In this series, CD10 (27/36) and vimentin were expressed more frequently. Expression of the cell differentiation marker CD10 was remarkably similar in all uterine sarcoma groups [26]. Recently, Oliva et al. [27] reported that although only 1 of 10 cases of endometrial stromal tumors were CD10-positive, four out of four high-grade ESSs in the study by Agoff et al. [28] and four of six high-grade ESSs in the study by McCluggage et al. [9], were CD10-negative, indicated that in high-grade ESS, decreased expression may be related to tumor differentiation. Our study consisted of 63 cases of ESS, out of which 13 progressed into either pelvic recurrence or pulmonary metastases, or both. Whether CD10-positive patients in our ESS cases truly represent a longer OS or independent prognostic factor cannot be ascertained, but CD10 did demonstrated significant association with the tumor’s classification (*P* = 0.001) and recurrence (*P* = 0.003). In addition, CD10-positive patients showed a longer DFS (*P* = 0.02). Although the level of CD10 (*P* = 0.033) expression appears to be a useful prognostic factor in univariate analysis, the limited range of our sample series did not permit any definitive conclusions. CD10 may represent a molecular marker that correlates with prognosis of ESS patients. Other markers have also been studied, for example, metalloproteinases, c-KIT, and CD34, all of which proved been negative [29].

In conclusion, the results of the present study indicate that FIGO stage and surgical procedure represent prognostic factors for patient survival, thus total hysterectomy + bilateral salpingo-oophorectomy followed by pelvic lymphadenectomy would likely result in an improved treatment outcome, representing a factor worth considering in the staging of surgery for ESS. CD10-negative status may contribute to the malignant characteristic and recurrence associated with ESS, and may play a significant role in molecular-targeted therapy.
